# Confined Quantum Hard Spheres

**DOI:** 10.3390/e23060775

**Published:** 2021-06-18

**Authors:** Sergio Contreras, Alejandro Gil-Villegas

**Affiliations:** División de Ciencias e Ingenierías, Campus León, Universidad de Guanajuato, Loma del Bosque 103, Lomas del Campestre, León, Guanajuato 37150, Mexico; s.contrerasarredondo@ugto.mx

**Keywords:** hard spheres, perturbation theory, quantum fluids, quantum Monte Carlo

## Abstract

We present computer simulation and theoretical results for a system of *N* Quantum Hard Spheres (QHS) particles of diameter σ and mass *m* at temperature *T*, confined between parallel hard walls separated by a distance Hσ, within the range 1≤H≤∞. Semiclassical Monte Carlo computer simulations were performed adapted to a confined space, considering effects in terms of the density of particles $\rho^{*}=N/V$, where *V* is the accessible volume, the inverse length $H^{-1}$ and the de Broglie’s thermal wavelength $\lambda_{B}=h/\sqrt{2\pi mkT}$, where *k* and *h* are the Boltzmann’s and Planck’s constants, respectively. For the case of extreme and maximum confinement, $0.5<H^{-1}<1$ and $H^{-1}=1$, respectively, analytical results can be given based on an extension for quantum systems of the Helmholtz free energies for the corresponding classical systems.

## 1. Introduction

The behavior of confined fluids have received considerable interest in recent years due to the research on microfluidic applications. Phase transitions that occur in pores are different to the ones observed in bulk systems, as has been determined in several studies of water in nanochanels [1]. Hydrogen in nanopores exhibits important quantum effects even at room temperatures [2]. The study of confined models is of great interest due to their multiple applications. Previous studies have found that the behaviour of a classical system of Lennard–Jones (LJ) particles confined between parallel walls separated by a distance Hσ, where σ is the size parameter of the LJ pair potential u(r) where u(σ)=0, presents a reduction in their critical point, with a clear asymptotic tendency to a two-dimensional LJ fluid when the separation between walls is reduced to maximum confinement [3]. In the case of the liquid–solid transition, the thermodynamic melting temperature of LJ system confined between parallel walls is higher than for the bulk case ($H^{-1}=0$) [4]. Schmidt and Löwen [5] studied the phase diagram of a hard-spheres (HS) system confined between parallel hard walls, and found a very rich formation of novel phases.

The case of quantum systems confined in slit pores has been studied previously for the HS model by Liu et al. [6]. In previous work [7] we have addressed the behavior of quantum fluids under confinement, considering quantum LJ particles (QLJ) confined between parallel hard walls, and introducing quantum corrections at the level of the Wigner–Kirkwood theory [8,9], with interesting findings in the way that the reduction of dimensionality from bulk (3D) to extreme confinement (2D) gives rise to the coupling of criticality with the pore size and de Broglie’s thermal wavelength $\lambda_{B}=h/\sqrt{2\pi mkT}$, where *k* and *h* are the Boltzmann’s and Planck’s constants, respectively, and *m* is the mass of a particle. From the fundamental solution of the Schrödinger equation, the behaviour of confined *D*-dimensional hard spheres has been determined for low-density systems [10] based on the assumption that diluted interacting quantum gases can be described as an ideal quantum gas interacting with small excluded spheres randomly distributed, i.e., a kind of an effective Bernoulli model of an ideal gas. A closed related-approach has also been used for classical hard particles [11,12], providing a route to construct equations of state for classical fluids [13]. Effects of confinement in quantum gases have also been studied in detail previously [10,14,15], where contributions to the free energy are functions of the size of confinement, the geometry and the connectivity or number of holes inside the container. What is clear is that the thermodynamics of dense quantum fluids become more elaborate with a complex dependency on the geometry and nature of the container, the thermal wavelength $\lambda_{B}$, density, temperature and spin.

The standard way for dealing with the prediction of structural and thermodynamic properties of fluids contained within pores is using density functional theory (DFT). In this paper we follow a different approach, with the motivation of introducing thermodynamic perturbation theory for confined fluids. We present Monte Carlo computer simulation results in the Canonical (NVT) and Isothermal–Isobaric (NPT) ensembles of quantum hard hard spheres (QHS) confined between hard planar walls separated by a distance Hσ, based on a semiclassical approach developed by Yoon and Scheraga for bulk fluids [16] and that we have adapted to confined systems. In the cases of maximum ($H^{-1}=1$) and extreme ($0.5<H^{-1}<1$) confinement we obtain analytical expressions for the Helmholtz free energy based on the approach given by Franosch et al. [17].

## 2. Method

If we consider a system of *N* quantum hard-sphere particles whose potential energy is $U_{HS}\left(r^{N}\right)$, then the partition function neglecting exchange effects is given by

(1) $$Z_{QHS}=\frac{1}{N!}\int W\left(r^{N}\right)dr^{N},$$

where $W(r^{N})$ is the Slater sum defined as [18]

(2) $$W\left(r^{N}\right)=\sum_{n}e^{-\beta E_{n}}\Psi_{n}^{*}\Psi_{n,}$$

where $E_{n}$ is the energy for the system’s quantum states whose stationary wave function is $\Psi_{n}$, according to the Schrödinger equation, $\hat{H}$,

(3) $$\hat{H}\Psi_{n}=E_{n}\Psi_{n},$$

where $\hat{H}$ is the Hamiltonian operator. In the classical limit,

(4) $$W\left(r^{N}\right)=W_{c}\left(r^{N}\right)=\lambda_{B}^{-3N}e^{-\beta U_{HS}\left(r^{N}\right)}$$

whereas in the semiclassical approximation $\lambda_{B}<\sigma$ and the Slater sum of the system can be expressed as [19,20]

(5) $$W\left(r^{N}\right)=W_{c}\left(r^{N}\right)W_{q}\left(r^{N}\right)$$

where $W_{q}\left(r^{N}\right)$ gives the quantum correction to the classical value.

The Slater sum $W_{q}\left(r^{N}\right)$ can be be expressed as a Zwanzig expansion [21] in terms of classical ensemble averages of the quantum Ursell functions $U_{n}$, considering now the quantum version of these functions, obtained through the solution of the Schrödinger equation. In the two-particle cluster approximation,

(6) $$W_{q}\left(r^{N}\right)=1+\sum_{i<j}U_{2}\left(i,J\right)$$

where

(7) $$U_{2}\left(i,J\right)=-e^{-\xi_{ij}^{2}}$$

with

(8) $$\xi_{ij}^{2}=2\pi {\left[\frac{r_{ij}^{*}-1}{\lambda_{B}^{*}}\right]}^{2}.$$

where $\lambda_{B}^{*}=\lambda_{B}/\sigma$ and $r_{ij}^{*}=|r_{i}-r_{j}|/\sigma$ is the reduced distance between particles *i* and *j* with positions $r_{i}$ and $r_{j}$, respectively. The two-particle Slater function is then given by

(9) $$w\left(i,j\right)=1+U_{2}\left(i,j\right)$$

Using Equations (1), (4) and (5) we can derive a semiclassical partition function for the QHS system, that follows the same mathematical structure as the Zwanzig perturbation theory for classical fluids [22],

(10) $$Z_{QHS}=\frac{1}{N!\lambda_{B}^{3N}}\int e^{-\beta U_{HS}\left(r^{N}\right)}W_{q}\left(r^{N}\right)dr^{N}$$

and introducing the classical HS partition function, $Z_{HS}=\lambda_{B}^{-3N}\int e^{-\beta U_{HS}\left(r^{N}\right)}dr^{N}/N!$, then

(11) $$\begin{array}{ccc} Z_{QHS} & = & Z_{HS}\frac{\int e^{-\beta U_{HS}\left(r^{N}\right)}W_{q}\left(r^{N}\right)dr^{N}}{\int e^{-\beta U_{HS}\left(r^{N}\right)}dr^{N}}\\ & = & Z_{HS}<W_{q}\left(r^{N}\right){>}_{HS} \end{array}$$

where $<\ldots {>}_{HS}$ denotes an ensemble average with respect to the classical HS system. The corresponding Helmholtz free energy $A_{QHS}=-kT\ln Z_{QHS}$ is then obtained, using Equations (9) and (11),

(12) $$\begin{array}{ccc} A_{QHS} & = & -kT\ln Z_{HS}-kT\ln\left[1+<\sum_{i<j}U_{2}\left(i,j\right){>}_{HS}\right]\\ & = & A_{HS}-kT<\sum_{i<j}U_{2}\left(i,j\right){>}_{HS} \end{array}$$

Last expression can be interpreted as the perturbation expansion for a classical system of particles interacting with the pair potential $u_{HS}\left(i,j\right)-kTU_{2}\left(i,j\right)$, where $u_{HS}\left(i,j\right)$ is the HS binary potential for particles *i* and *j* and $-kTU_{2}\left(i,j\right)<1$ is the perturbation potential. Additionally,

(13) $$\begin{array}{ccc} <\sum_{i<j}U_{2}\left(i,j\right){>}_{HS} & = & \frac{N(N-1)}{2}<U_{2}\left(i,j\right){>}_{HS}\\ & \approx & \frac{N^{2}}{2V}<U_{2}\left(r\right){>}_{HS} \end{array}$$

where *r* is the relative position between two particles. Consequently, Equation (12) can be rewritten as

(14) $$\frac{A_{QHS}}{NkT}=\frac{A_{HS}}{NkT}+\frac{\beta \rho }{2}<-kTU_{2}\left(r\right){>}_{HS}$$

that corresponds to the first-order perturbation expansion of the Helmholtz free energy for a QHS system, in terms of its classical counterpart as reference system, with a mean-energy term for the potential $-kTU_{2}\left(r\right)$. An alternative expression for $A_{QHS}$ was developed by Nordholm and coworkers [23,24], where quantum effects were included in the partition function of *N* independent HS particles, where packing behaviour was modelled as if one particle is enclosed in a cage induced by the remaining N−1 particles. This excluded a volume mechanism can be described quantum mechanically using the standard particle-in-a-box solution of wave mechanics.

In the MC simulation scheme given by Yoon and Scheraga [16], Equation (14) is used rewritten in terms of the two-body Slater sum w(r), and the approximation $U_{2}\left(r\right)\approx \ln\left(1+U_{2}\left(r\right)\right)=\ln w\left(r\right)$, i.e.,

(15) $$\frac{A_{QHS}}{NkT}=\frac{A_{HS}}{NkT}+\frac{\beta \rho }{2}<-kT\ln w\left(r\right){>}_{HS}$$

Introducing an effective Boltzmann factor,

(16) $$w\left(i,j\right)=e^{-\beta \Gamma_{ij}}=1-e^{-\xi_{ij}^{2}},$$

the semiclassical MC simulations are performed with the standard classical MC method, using $\Gamma_{ij}$ as an equivalent energy and the Slater sum as the quantum-mechanical probability instead of the Boltzmann one.

Quantum hard spheres follow Boltzmann statistics since exchange effects decay exponentially to zero in harsh repulsive systems at temperatures where de Broglie thermal wavelengths are comparable to the size of particles, and then quantum diffraction effects are not negligible. In the case of solid phases, previous studies have demonstrated that thermodynamic and structural properties of quantum systems are basically unaltered by modifying the type of statistics (Bose-Einstein, Fermi-Dirac or Boltzmann-Gibbs) [25]. Chandler and Wolynes [26] have demonstrated by a path-integral approach that exchange effects can be described as associating ring molecules, using the exact isomorphism between quantum theory and classical statistical mechanics of polyatomic ring fluids. In a first approximation, considering only dimer association at low temperatures and liquid densities, the hard-core repulsion of a QHS system with $\lambda_{B}<\sigma$ reduces the association constant, with respect to its ideal-gas value, by a factor of the order of $\tau \approx 2e^{-2\pi \sigma^{2}/\lambda_{B}^{2}}$. In this work we are considering systems with $\lambda_{B}\leq 0.6\sigma$, that correspond to $\tau \leq 2\times 10^{-8}$, i.e., the majority of the ring molecules will be non-associated since the population of dimers is extremely low, an indication that in the semiclassical approach that we are following here we can safely neglect exchange effects even at high densities.

## 3. Results

Computer simulations were obtained using the method described in the previous section, applying the standard Metropolis algorithm [27] with a random sampling technique, for QHS particles confined between parallel hard walls, and considering the particle–wall interaction

(17) $$u\left(r\right)=\left\{\begin{array}{c} \infty \;if\;\left|z\right|<\sigma /2,\\ 0\;if\;\left|z\right|\geq \sigma /2, \end{array}\right.$$

where *z* is the distance between the fluid particles and the walls, and usual periodic boundary conditions were applied in the *x* and *y* coordinates. Simulations were performed using 512 QHS particles, for densities $\rho^{*}=0.1,\;0.2,\;0.3,\;0.4,\;0.5,\;0.6,\;0.7$ and thermal wavelengths $\lambda_{B}^{*}=0.0,\;0.1,\;0.2,\;0.3,\;0.4,\;0.5,\;0.6$. The total number of movements for equilibration and averaging were $3\times 10^{8}$ and $1\times 10^{8}$, respectively, with an overall acceptance of 40% average. Wall separations were taken from H=1.0 up to 5.0 in steps of 0.2, and H=6.0 up to 11, in steps of 1.0.

### 3.1. Confined QHS Global Behaviour

When we consider the confinement effect due to parallel hard walls, the Helmholtz free energy of the system can be expressed as

(18) $$\frac{A_{CQHS}}{NkT}\left(\rho^{*},\lambda_{B}^{*},H^{-1}\right)=\frac{A_{CHS}}{NkT}\left(\rho^{*},H^{-1}\right)+\frac{A_{CQ}}{NkT}\left(\rho^{*},\lambda_{B}^{*},H^{-1}\right)$$

where $A_{CHS}$ is the Helmholtz free energy of confined classical hard spheres and $A_{CQ}$ is the contribution due to the quantum nature of the system. In the classical approximation, i.e., $\lambda_{B}^{*}=0$, $A_{CQ}=0$. On the other hand, for the bulk case, i.e., $H^{-1}=0$, $A_{CHS}\left(\rho^{*},H^{-1}=0\right)=A_{HS}\left(\rho^{*}\right)$, and Equation (18) reduces to the Helmholtz free energy of the bulk system,

(19) $$\frac{A_{CQHS}}{NkT}\left(\rho^{*},\lambda_{B}^{*},H^{-1}=0\right)=\frac{A_{HS}}{NkT}\left(\rho^{*}\right)+\frac{A_{CQ}}{NkT}\left(\rho^{*},\lambda_{B}^{*},H^{-1}=0\right)$$

where $A_{HS}\left(\rho^{*}\right)$ can be obtained from the Carnahan–Starling equation of state [28],

(20) $$\frac{A_{CQHS}}{NkT}\left(\rho^{*},\lambda_{B}^{*},H^{-1}=0\right)=\frac{A_{ideal}^{3D}}{NkT}+\frac{4\eta_{e}-3\eta_{e}^{2}}{{\left(1-\eta_{e}\right)}^{2}}$$

where $A_{ideal}^{3D}$ is the ideal contribution and

(21) $$\eta_{e}/\eta =1+p_{1}\lambda_{B}^{*}+p_{2}\lambda_{B}^{*2},$$

where $p_{1}=0.588868$ and $p_{2}=0.524248$ are parameters obtained by fitting to the NVT-MC values.

**Figure 1:**
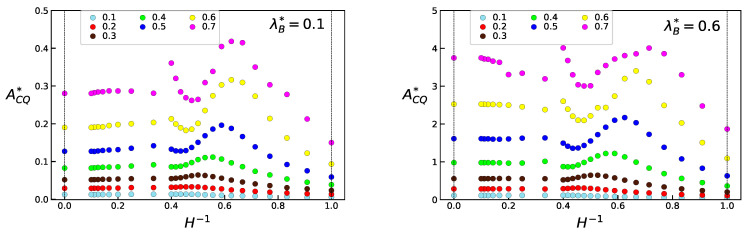
Helmholtz free energy $A_{CQ}^{*}=A_{CQ}/NkT$ for the confined QHS system, obtained from theoretical results and simulations, as a function of the inverse wall separation $H^{-1}$, for thermal wavelengths $\lambda_{B}^{*}=0.1$ and 0.6.

**Figure 2:**
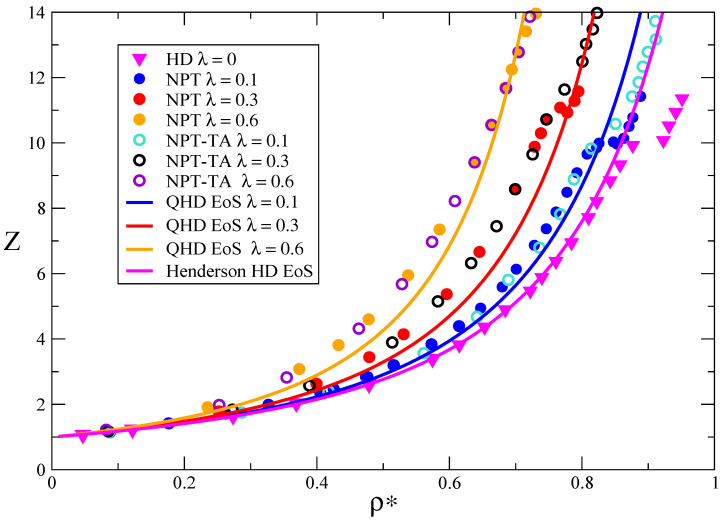
Compressibilty factor *Z* of quantum hard disks. Computer simulation results are given as solid symbols (standard NPT MC) and open symbols (NPT Test-Area MC) for different thermal wavelengths $\lambda_{B}^{*}$, including the classical system ($\lambda_{B}^{*}=0$). Theoretical results according to the classical [29] and quantum version of the Henderson equation of state are also reported.

For the general case when $H^{-1}\neq 0$ we can determine $A_{CQ}$ from NVT-MC simulations, as can be seen in Figure 1. We observe that, for all thermal wavelengths, two different ranges are observed where $A_{CQ}$ behaves differently. When $0\leq H^{-1}<0.4$, the free energy contribution due to quantum effects and confinement varies slowly with respect to the bulk phase ($H^{-1}=0$). When $0.4<H^{-1}\leq 1$, $A_{CQ}$ has strong variations with $H^{-1}$ for densities $\rho^{*}>0.4$. It is convenient to discriminate a region for $0.5<H^{-1}<1$, that will be characterised as of extreme confinement that ends with the case of maximum confinement, $H^{-1}=1$, when the distance between walls is equal to a HS diameter. In this limit, the behaviour of the system can be approximated by a hard disks system. Following the same approach used for bulk QHS, the Helmholtz free energy for quantum hard disks (QHD) is obtained from the equation of state derived by Henderson for classical hard disks [29] as a function of a 2D effective packing fraction $\gamma_{e}$, in order to reproduce the NVT-MC simulated values,

(22) $$\frac{A_{CQHS}}{NkT}\left(\rho^{*},\lambda_{B}^{*},H^{-1}=1\right)=\frac{A_{ideal}^{3D}}{NkT}+\frac{9\gamma_{e}}{8(1-\gamma_{e})}-\frac{7}{8}\ln\left(1-\gamma_{e}\right)$$

where

(23) $$\gamma_{e}/\gamma =1+q_{1}\lambda_{B}^{*}+q_{2}\lambda_{B}^{*2},$$

and $q_{1}=0.35367$ and $q_{2}=0.224205$. For this equation we are considering that $\rho_{2D}=N/A_{s}$ is a 2D density for *N* particles distributed along a surface with area $A_{s}$, and γ is the actual 2D packing fraction of the system,$\gamma =\pi \rho_{2D}\sigma^{2}/4$. The theoretical prediction for the compressibility factor for QHD systems, $Z_{QHD}=\beta P/\rho$, is presented in Figure 2, compared with NPT-MC simulation values, obtained from a standard NPT algorithm and also applying the Test Area MC method [30]. From these results it is clear that the introduction of quantum effects increases the values of the pressure with respect to the classical case, and the liquid–solid transition occurs at lower densities when $\lambda_{B}$ increases. Since it is well known that the quantum correction to hard particles increases its effective size, we can expect this behaviour. However, the nature of the transition as well the presence of a stable hexactic phase requires a more detailed study.

**Figure 3:**
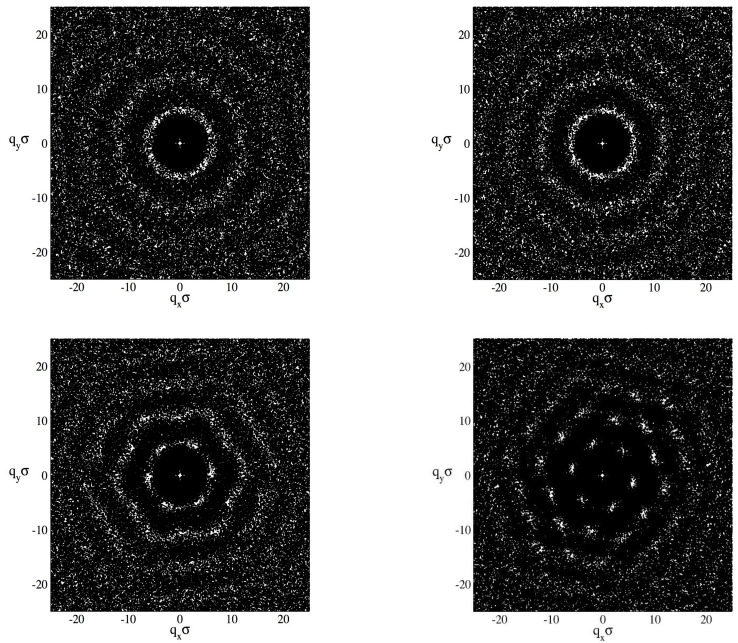
Structure factor for QHS in maximum confinement *H* = 1 and density ρ* = 0.8. The values of the thermal wavelength are $\lambda^{*}=0.0$ and $\lambda^{*}=0.1$ (top-left and top-right figures, respectively); $\lambda^{*}=0.2$ and $\lambda^{*}=0.6$ (bottom-left and bottom-right figures, respectively).

The behaviour of the structure factor of QHS at maximum confinement is presented in Figure 3. The transition from the liquid to the solid phases is given for the same density, $\rho^{*}=0.8$, varying $\lambda_{B}^{*}$. The system evolves from a liquid phase ($\lambda_{B}^{*}=0$) up to a solid phase ($\lambda_{B}^{*}=0.6$) and in between a hexactic-like phase can be observed for $\lambda_{B}^{*}=0.2$. This feature is consistent with the phase diagram for QHD given in Figure 1, since clearly for $\lambda_{B}^{*}\approx 0.1$ the liquid–solid transition boundary has shifted to densities equal to 0.8.

The clear discontinuity observed for $A_{CQ}$ that arises around $H^{-1}=0.4$ at high densities is consistent with the observation of a corresponding strong freezing transition in the phase diagram for confined classical HS around $H^{-1}=0.5$ [5]. By the same effect observed in Figure 2 for hard disks, since the particles swell by increasing the thermal wavelength $\lambda_{B}^{*}$ we can expect that the corresponding transitions will appear at higher values of *H* than in the classical state.

### 3.2. Equation of State for Extreme Confinement

For the case of extreme confinement, we follow the same procedure used for QHS and QHD, i.e., to consider an analytical expression for classical HS and then to map onto a quantum expression by considering an effective density.

Franosch et al. [17] derived an expression for the Helmholtz free energy of classical HS confined between two parallel hard walls, for the case 1≤H≤2, defining the confinement parameter L=H−1,

(24) $$\frac{A_{CHS}}{NkT}\left(\rho^{*},L\right)=\frac{A_{ideal}^{3D}}{NkT}+\frac{A_{HD}}{NkT}\left(\rho^{*},L\right)+\frac{\Delta A}{NkT}\left(\rho^{*},L\right)$$

where $A_{ideal}^{3D}$ is the 3D ideal term, $A_{HD}$ is the free energy for hard disks of diameter $\sigma_{L}={\left(\sigma^{2}-L^{2}\right)}^{1/2}$, and ΔA is the contribution arising from confinement, given by

(25) $$\frac{\Delta A}{NkT}=-\pi \rho \int g\left(r\right)\left[e^{-\beta V_{eff}\left(r;L\right)}-1\right]rdr,$$

where $V_{eff}\left(r;L\right)=-2kT\ln\left(1-\sqrt{\left(\sigma^{2}-r^{2}\right)/L^{2}}\right)$ and $g\left(r\right)$ are the radial pair distribution function of the hard-disk reference fluid of diameter $\sigma_{L}$. The integral involved in Equation (25) can be performed using the approximation $g\left(r\right)\approx g\left(\sigma_{L}\right)$ [17], and then obtaining

(26) $$\frac{\Delta A}{NkT}=\frac{5}{3}\gamma L^{2}g\left(\sigma_{L}\right).$$

**Figure 4:**
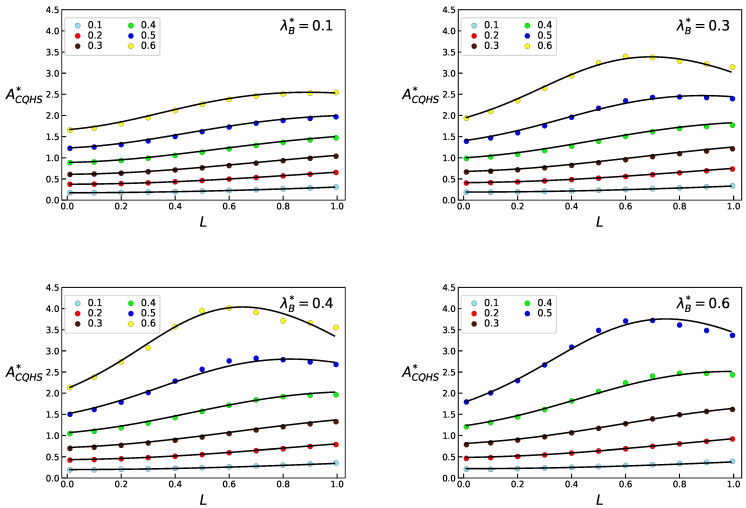
Helmholtz free energy $A_{CQHS}^{*}=A_{CQHS}/NkT$ for the confined QHS system, obtained from theoretical results and simulations, as a function of density, for thermal wavelengths $\lambda_{B}^{*}=0.1,0.3,0.4$ and 0.6.

**Figure 5:**
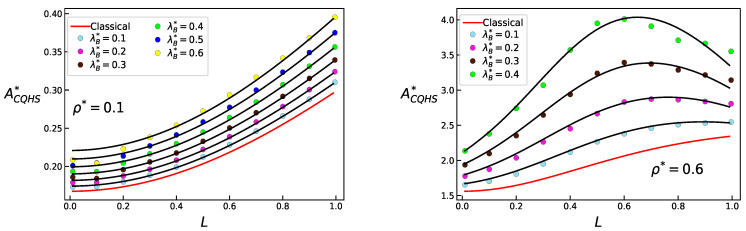
Helmholtz free energy $A_{CQHS}^{*}=A_{CQHS}/NkT$ for the confined QHS system, obtained from theoretical results and simulations, as a function of *L*, for densities $\rho^{*}=0.1$ and $\rho^{*}=0.6$.

The classical expression can be used to describe the contribution to the free energy of the CQHS system, where now the free energy is given in terms of the corresponding quantum expressions,

(27) $$\frac{A_{CQHS}}{NkT}\left(\rho^{*},\lambda_{B}^{*},H^{-1}\right)=\frac{A_{ideal}^{3D}}{NkT}+\frac{A_{QHD}}{NkT}\left(\rho^{*},\lambda_{B}^{*},H^{-1}\right)+\frac{\Delta A_{Q}}{NkT}\left(\rho^{*},\lambda_{B}^{*},H^{-1}\right)$$

and the free energy of the quantum hard disks of diameter $\sigma_{L}$ is given by the Henderson equation of state evaluated with an effective packing fraction that reproduces the NVT-MC simulation results for the confined system,

(28) $$\gamma_{Q}=\left(1+q_{1}\lambda_{B}^{*}+q_{2}\lambda_{B}^{*2}\right)+q_{3}\lambda_{B}^{*2},$$

where $q_{3}=\left(q_{31}+q_{32}\gamma \right)L+q_{33}\gamma^{2}L^{2}$, with $q_{31}=-0.40144$, $q_{32}=4.7426$ and $q_{33}=-7.95290$. Notice that in the limit L=0 we recover the expression given by Equations (22) and (23). In Figure 4 and Figure 5 results are given for $A_{CQHS}^{*}$ as functions of density, *L* and $\lambda_{B}$, as obtained from MC simulations and using the quantum version of the free energy of the confined system, Equations (27) and (28).

## 4. Discussion

In this work we have presented theoretical and computer simulation results for QHS particles confined between planar hard walls. Since the quantum nature of the system given by the thermal wavelength $\lambda_{B}^{*}$ introduces a new parameter in the statistical and thermodynamic properties of the confined system, its coupling with the confining parameter *H* increases the complexity of the phase behaviour already observed for confined classical hard spheres [5]. One of the main features observed in our study is the swelling effect of the hard spheres that modifies the phase diagram of the confined classical system, particularly relevant in the case of maximum confinement, where the liquid–solid transition of QHD occurs at lower densities when compared with classical hard disks. We also considered analytical expressions for the case of extreme confinement, $0.5<H^{-1}\leq 1$, based on expressions for the classical system. Similar to what has been already reported for the bulk system [16,31,32], these classical expressions are valid if the packing fraction is modified by considering the swelling effect of the quantum particles and the confinement parameter *H*. In a future communication we will explore this thermodynamic approach in order to obtain equations of state for a fluid confined in slit pores, based on perturbation theory, following the methods already developed for bulk systems [31,32].

## Data Availability

The data that support the findings of this article are available on request from the corresponding author.
